# Assessment of soil quality in an arid and barren mountainous of Shandong province, China

**DOI:** 10.1038/s41598-023-46136-6

**Published:** 2023-11-15

**Authors:** Lu Wang, Jianyao Guo, Xiumei Liu, Kun Li, Liang Ma, Yehan Tian, Jinming Wang, Qingdong Zhang, Yaozhen Tian, Chuanrong Li, Min Lu

**Affiliations:** 1 Landscape Architecture Research Center, Shandong Jianzhu University, Jinan, 250101 Shandong China; 2 State Forestry and Grassland Administration Key Laboratory of Silviculture in Downstream Areas of the Yellow River, Tai’an, 271018 Shandong China; 3 Shandong Forestry Protection and Development Service Center, Jinan, 250000 Shandong China; 4 Shanghai Environment College, Shanghai, 200135 China

**Keywords:** Ecology, Enzymes

## Abstract

Forest soils are important components of forest ecosystems, and soil quality assessment as a decision-making tool to understand forest soil quality and maintain soil productivity is essential. Various methods of soil quality assessment have been developed, which have occasionally generated inconsistent assessment results between soil types. We assessed the soil quality of five communities (herb, shrub, *Quercus acutissima*, *Pinus thunbergii*, and *Q. acutissima*–*P. thunbergii* mixed plantation) using two common methods of dry and barren mountains in the Yimeng Mountain area, China. Sixteen soil physical, chemical and biological properties were analysed. The soil quality index was determined using the established minimum data set based on the selection results of principal component analysis and *Pearson* analysis. Silt, soil total phosphorus (P), soil total nitrogen (N), L-leucine aminopeptidase, acid phosphatase and vector length were identified as the most representative indicators for the minimum data set. Linear regression analysis showed that the minimum data set can adequately represent the total data set to quantify the impact of different communities on soil quality (*P* < 0.001). The results of linear and non-linear methods of soil quality assessment showed that the higher soil quality index was *Pinus* forest (0.59 and 0.54), and the soil quality index of mixed plantation (0.41 and 0.45) was lower, which was similar to the herb community (0.37 and 0.44). Soil quality was mostly affected by soil chemical properties and extracellular enzyme activities of different communities, and the different reasons for the low soil quality of mixed plantations were affected by soil organic carbon (C) and total C. Overall, we demonstrate that the soil quality index based on the minimum data set method could be a useful tool to indicate the soil quality of forest systems. Mixed plantations can improve soil quality by increasing soil C, which is crucial in ecosystem balance.

## Introduction

Forests are the largest C repository in terrestrial ecosystems^1^. Comprehensively improving the stability of forest ecosystems and ecological service functions, and increasing forest utilization are effective ways to address current climate change. However, the problem of difficult use of forestland in dry and barren mountainous has become increasingly prominent. Severe rocky desertification^2^, and insufficient nutrient and water supplies have slowed plant growth and development^3,4^. Therefore, understanding the soil environment in mountainous areas will help reveal the impact of soil quality on sustainable forest management.

Soil is an important environment for regulating nutrient balance and plant growth and development in forest ecosystems^5^, and different forest types have different soil environments^6^. Soil quality is a comprehensive reflection of soil physical, chemical and biological characteristic^7^. Soil moisture and nutrients are crucial in the energy flow and transmission of the ecosystem, and they reflect the status of soil quality^8^. Soil extracellular enzymes are derived from soil microbial metabolic activities, plant root secretion and animal residue decomposition^9^. Extracellular enzyme activities can reflect the functional characteristics of soil microbes and participate in C, N and P. absorption and utilization in soil biochemical reactions^10^. The soil C-acquiring enzyme β-1,4-glucosidase(BG) can be used to catalyse the C cycle, the soil N-acquiring enzymes β-1,4-*N*-acetylglucosaminidase (NAG) and L-leucine aminopeptidase (LAP) are responsible for peptidoglycan and leucine decomposition, and the soil P-acquiring enzyme acid phosphatase (AP) can catalyse organophosphorus chemical mineralisation^11^. Previous studies have reported that the biological characteristics of forest soil quality are catalase activity (CAT), urease activity (UR) and protease activity (PR)^12–14^, but the four major acquisition enzymes of soil C, N and P and vector properties are rarely used for soil quality assessment. Therefore, the application of extracellular enzymes and vector properties is conducive to understanding the biological characteristics of the dry and barren mountainous ecosystem in the Yimeng Mountainous Area, China, and is critical in the impact of microbial metabolism on soil quality against the background of global climate change.

Soil quality cannot be measured directly, and a comprehensive assessment of soil indicators is needed to quantify soil quality^15^. Soil quality assessment methods include the soil quality index (SQI), soil productivity index model (PI), and system analysis method^16–18^. At present, the most used soil quality assessment method in agriculture and forestry is the SQI^19,20^, but there are many soil indicators involved, and it is difficult to obtain data on all soil properties, and information overlaps. Therefore, simplifying the evaluation indicators and constructing the minimum data set (MDS) for soil quality assessment^21^ could reduce the workload, reduce the cost, and prevent data redundancy^22,23^. Zhang et al.^24^ used the MDS to assess the soil quality at different stages of vegetation succession in China's karst areas, and the soil quality at the stage from farmland to secondary forest showed an increasing trend. Zhao et al.^25^ used the minimum data set to assess the different ages of *Larix principis-rupprechtii*, and the soil quality gradually improved within 16 to 44 years. Therefore, it is essential to establish an MDS to assess the soil quality of forest communities in a specific area.

We researched the soil parameters, and four physical properties, six chemical properties, and six biological properties were determined in five communities, combined with principal components and correlation analysis to establish a minimum data set. We compared linear and non-linear models for soil quality assessment and analysed the factors influencing soil quality in different communities of dry and barren mountains in the state-owned Dawa Forest Farm in the Yimeng Mountain Area, China.

Our aims were (1) to establish an MDS with a proper indicator for soil quality assessment through physical, chemical and biological characteristics; (2) to compare and analyse the applicable between linear and non-linear scoring method for soil quality assessment; and (3) to evaluate the soil quality of different communities using the SQI method and determine the affecting indicators. The results of this study can provide a more appropriate data reference, which is essential to realize the effective use of forest soil and the stability of the ecosystem.

## Materials and methods

### Study site

The sites were in the state-owned Dawa Forest Farm of the Yimeng Mountain area of Shandong province, China (latitude 35° 30′ 2.04″ to 35° 30′ 24.43″ N and longitude 117° 55′ 44.14″ to 117° 56′ 3.36″). The mean annual temperature of this region is 12.8 ℃, the mean annual precipitation is 600 mm. The study site has a warm temperate continental monsoon climate, and a frost-free period 191 d. The sediment is dominated by gneiss, and brown forest soil, the average thickness of the arbor soil layer is approximately 20 cm.

Five typical communities with consistent slope orientation, slope position, altitude, and growth were selected as study plots in October 2019: herb, shrub, *Quercus acutissima*, *Pinus thunbergii*, and *Quercus acutissima*–*Pinus thunbergii* mixed-plantation plots. The forest plots were 30-year-old plantations, the main species of vegetation in this area are *Quercus acutissima*, *Pinus thunbergii*, *Vitex negundo*, *Cymbopogon goeringii*, *Achnatherum pekinense*, *Viola collina*, *Conyza canadensis*, and *Artemisia stechmanniana*.

### Experimental design and soil sampling

Three plots of 5 × 5 m were established in the herb community, three plots of 10 × 10 m were established in the shrub community, and three plots of 30 × 30 m were established in each of the *Quercus acutissima*, *Pinus thunbergii*, and mixed-plantation communities. The spaces between adjacent plots were at least 10 m. In each plot, soil samples were collected using a soil auger (diameter, 4 cm) from the 0–15 cm soil layer at 15 random points and then mixed into a composite sample as one replicate. Samples were collected from a total of 225 random points across the different sites, with three plots per community as three independent replicates. In total, 15 composite samples were established^26^. A subsample of each composite sample was immediately placed in an ice box, transported to the laboratory, and then stored at 4 °C for the analysis of extracellular enzyme activities within two weeks. The other subsample was air-dried for physicochemical analysis (Table 1).

**Table 1:** Methods used in laboratory analyzes for selected indicators, Particle size: sand, silt and clay, extracellular enzyme: β-1,4-glucosidase(BG), β-1,4-*N*-acetylglucosaminidase (NAG), L-leucine aminopeptidase (LAP) and acid phosphatase (AP).

Indicator	Method
Soil moisture	Drying method
pH	Glass-electrode meter method (FiveEsay Plus, Mettler Toledo, Schwarzenbach, Switzerland)
Particle size	Laser particle size metre method (Malvern Instruments, Malvern, England)^27^
Organic C	Titration method (Top Burret M, Eppendorf AG, Hamburg, Germany)^28^
Available P	Colorimetric method (UV2300, Hitachi, Shanghai, China)^29^
Total C and N	Element analyser method (ECS4010, Costech, Milan, Italy)
Total P	Colorimetric method(UV2300, Hitachi, Shanghai, China)^30^
Extracellular enzyme	Microporous plate fluorescence method (Synergy MX BioTek, Vermont, USA)^31^

### Soil quality index

Evaluation steps of SQI: (1) *Pearson* conducts correlation analysis of soil indicators, PCA was used to group the indicators, and the component with eigenvalue ≥ 1 was selected. The indicators with loadings ≥ 0.5 in the same component were classified into one group. If the loadings of one indicator in different components were ≥ 0.5, the indicator was classified into the group where the indicator had the lowest correlations with other indicators, the total data set was built, and the norm value of each group of indicators was calculated. (2) The indicator whose norm value was within the 10% range of the maximum total value of a group was selected for further correlation analysis. If the indicators were significantly correlated, then the indicator with the highest norm value was retained in the minimum data set, and all others were eliminated. The noncorrelated indicators were considered important and retained in the minimum data set in the same group. (3) After determining the minimum data set for the soil quality index, each soil indicator was transformed into unit-less scores ranging from 0.00 to 1.00 using linear and non-linear scoring function methods^30,32^.

The Norm Eq. (1) was used as follow:

1 $${N}_{ik}=\sqrt{{\sum }_{j=1}^{k}\left({u}_{ik}^{2}{e}_{k} \right)}$$

where *N*_*ik*_ is the norm value of k PCs with eigenvalues ≥ 1 for variable *i*, *u*_*ik*_ is the loading of soil variable* i* in component *k*, and *e*_*k*_ is the eigenvalue of component *k.*

The following Non-linear curves were used as sigmoidal type equation Eq. (2) scoring functions:

2 $${S}_{NL}=\frac{a}{1+{(x/{x}_{0})}^{b}}$$

where a is the maximum value (defined as a = 1 in this study) reached by the function,* x* is the value of the selected indicator and *x*_*0*_ is the mean value of each indicator corresponding to the soils. *b* is the slope of the equation and was set as − 2.5 for “more is better” and + 2.5 for “less is better” functions.

The following linear curves were subsequently used as “more is better” Eq. (3) or “less is better” (Eq. (4)) scoring functions:

3 $${S}_{L}=\frac{{y}_{i}-{y}_{mi n}}{{y}_{max}-{y}_{min}}$$

4 $${S}_{L}=1-\frac{{y}_{i-}-{y}_{min}}{{y}_{max}-{y}_{min}}$$

where *y*_*i*_ is the measured index value, *y*_*min*_ is a soil variable of the minimum value, and *y*_*max*_ is a soil variable of the maximum value

5 $$\mathrm{SQI}=\sum_{i=1}^{n}{w}_{i}{s}_{i}$$

where SQI is the comprehensive soil quality score index, *W*_*i*_ is the weight of the *i*th evaluation indicator, *s*_*i*_ is the index score, and *n* is the number of evaluation indicators.

### Statistical analysis

Vector length, representing microbial C limitation, was calculated as the square root of the sum of (lnBG/ln(NAG + LAP))^2^ and (lnBG/lnAP)^2^ (Eq. (6)). The vector angle, representing microbial N or P limitation, was calculated as the arctangent of the line extending from the plot origin to point (lnBG/lnAP, lnBG/ln(NAG + LAP) Eq. (7))^31^. The equations are as follows:

6 $$\mathrm{Vector length}=\sqrt{{\left(lnBG/\mathrm{ln}[NAG+LAP]\right)}^{2}+{\left(lnBG/lnAP\right)}^{2}}$$

7 $$\mathrm{Vector angle}=Degrees\left(ATAN2\left(lnBG/lnAP\right),\left(lnBG/ln\left[NAG+LAP\right]\right)\right)$$

The mean and standard deviation were calculated using SPSS Statistics 22 software. One-way analysis of variance and Duncan analysis were used to compare the differences between communities. The significance test was carried out at alpha = 0.05. Excel was used to process the data. *Pearson* correlation was used to analyse the correlation between soil index variables. Principal component analysis (PCA) was used to simplify the data analysis, reduce the dimensionality of complex data, and solve the problem of multicollinearity among explanatory variables. Linear regression analysis determined the relationship between the minimum data set and the total data set. All bar graphs were drawn using Origin 2018.

## Results

### Establishment of the minimum data set

**Figure 1:**
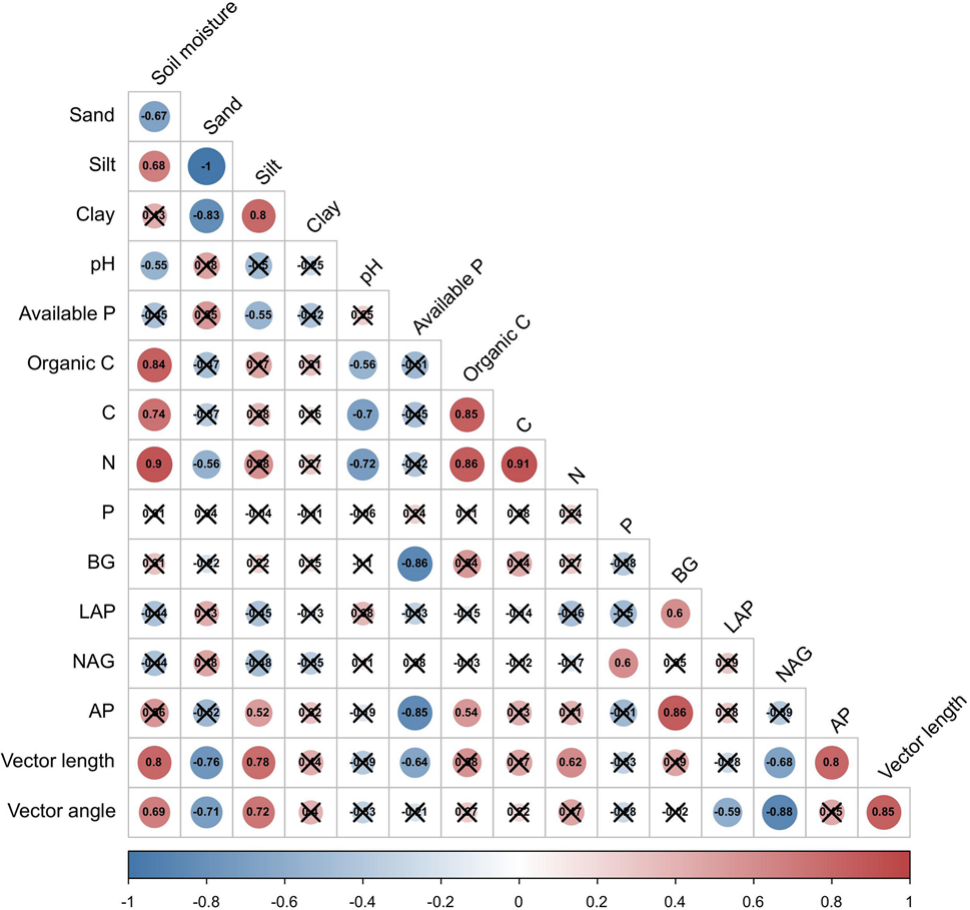
Correlation heatmap between the soil physicochemical properties, soil enzyme activities, and soil microbial nutrient limitation (*P* < 0.05). The value of the correlation coefficient is displayed in the circle in the figure, red represents a positive correlation, blue represents a negative correlation, the shade of the colour represents the strength of the correlation, and the × in the figure represents no significant correlation.

As a result, the indicators were determined with eigenvalues ≥ 1 in four PCs, which accounted for over 90.883% of the variation in the soil characteristics. This agreed with the requirements of information extraction (Table 2). The 30.255% variation was due to the first PC1, and more than 20% of the variation was due to PC2 and PC3, while PC4 accounted for 18.959% of the variation. The weight values from the TDS indicators were relatively low and similar. In general, indicators with absolute factor loading values ≥ 0.50 were considered highly weighted PCA indicators, which could be first selected from each PC, and divided into 5 groups according to the PC results. *Pearson* correlation analysis was used to check the correlation between these indicators to reduce redundancy (Fig. 1). The minimum data set (MDS) selected indicators whose Norm value was within 10% of the highest value of the group and eliminates them with strong correlation. Finally, the MDS related indicators were determined as silt, total N, total P, LAP, AP, and vector length.

**Table 2:** Principal component analysis results and standard values of 16 soil properties and soil quality indicator weights in the minimum data set.

Indicator	PC1	PC2	PC3	PC4	Communality	Grouping	Norm	TDS Weight
Soil moisture	0.813	0.077	0.332	0.372	0.916	1	2.001	0.063
Sand	− 0.358	− 0.058	− 0.874	− 0.301	0.986	3	1.856	0.068
Silt	0.377	0.052	0.859	0.313	0.980	3	**1.857**	0.067
Clay	0.053	0.119	0.903	0.098	0.841	3	1.673	0.058
pH	− 0.732	0.089	− 0.205	− 0.057	0.590	1	1.664	0.041
Available P	− 0.314	− 0.781	− 0.435	− 0.033	0.899	2	1.775	0.062
Organic C	0.867	0.3	0.186	− 0.018	0.877	1	2.014	0.060
C	0.921	0.23	0.022	− 0.017	0.903	1	2.070	0.062
N	0.958	0.005	0.224	0.101	0.978	1	**2.154**	0.067
P	0.286	− 0.542	0.117	− 0.668	0.834	5	**1.667**	0.057
BG	0.275	0.945	0.078	− 0.01	0.975	2	1.837	0.067
LAP	− 0.4	0.802	− 0.295	− 0.232	0.943	2	**1.838**	0.065
NAG	− 0.009	0.046	− 0.229	− 0.952	0.962	4	1.712	0.066
AP	0.317	0.778	0.261	0.411	0.943	2	**1.803**	0.065
Vector length	0.488	0.309	0.429	0.647	0.937	4	**1.831**	0.064
Vector angle	0.31	− 0.156	0.411	0.829	0.978	4	1.787	0.067
Eigenvalue	4.841	3.344	3.323	3.034	–	–	–	–
Variance (%)	30.255	20.901	20.767	18.959	–	–	–	–
Cumulative variance (%)	30.255	51.156	71.923	90.883	–	–	–	–

Boldface loading values correspond to those selected from the Norm for correlation analysis and the soil indicators included in the minimum date set. Calculated weights for the indicators in the minimum date set. Clay: soil granularity < 0.002 mm; Silt: soil granularity 0.002–0.05 mm and Sand: soil granularity 0.05–2 mm.

*PC* principal component; *BG* β-1,-4-glucosidase; *LAP* leucine aminopeptidase; *NAG* β-1,4-*N*-acetylglucosaminidase; *AP* alkaline phosphatase; *TDS* total date set.

### Scores and weights of the MDS indicators

**Figure 2:**
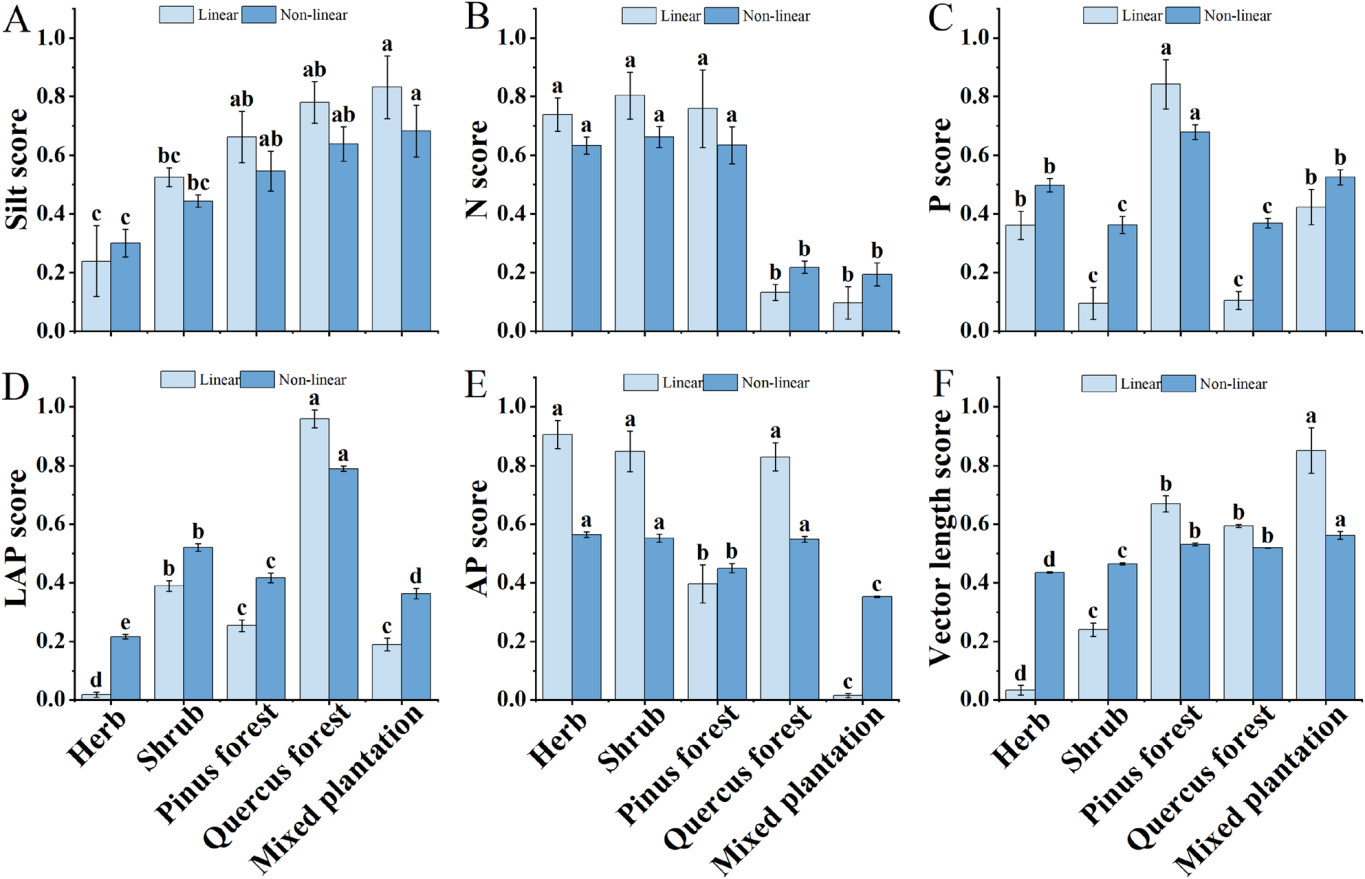
Linear and non-linear score values of the MDS; values are the means ± standard error of the mean. The different letters indicate significant differences between treatments according to the Tukey test; lowercase letters (such as a, b, c, d) indicate significant differences (*P* < 0.05) among the five communities. (**A**–**C**): soil physicochemical properties score; (**D**,**E**): soil enzyme activity score; (**F**): soil microbial nutrient limitation.

There were significant differences (*P* < 0.05) between linear and non-linear scoring of the minimum data set indicators for different communities (Fig. 2). The score values of the silt indicator ranged from 0.23 to 0.83 and 0.30 to 0.68 for the linear and non-linear models, respectively. The linear and non-linear total N score values followed the order shrub > *Pinus* forest > herb > *Quercus* forest > mixed plantation, and there was no significant difference between the *Quercus* forest and mixed plantation. The score values of total the P indicator ranged from 0.09 to 0.84 and 0.36 to 0.68 for the linear and non-linear models, respectively. The score values of the total LAP indicator ranged from 0.02 to 0.95 and 0.22 to 0.79 for the linear and non-linear models, respectively. The linear and non-linear AP score values followed the order herb > shrub > *Quercus* forest > *Pinus* forest > mixed plantation, while the linear and non-linear vector length scores followed the order mixed plantation > shrub > *Pinus* forest > *Quercus* forest > *shrub* > herb. Generally, the linear scores of the MDS indicators were consistent with the non-linear scored ranking results of the five communities.

In the non-linear model, total N, total P, LAP, and AP were the more the better indicators; silt and vector length were the less the better indicators (Table 3). In the linear function, total N, total P, LAP, and AP were calculated by Eq. (3) to calculate the soil quality index, and silt and vector length were calculated by Eq. (4) to calculate the soil quality index. Vector length had the highest weighting that resulted in the highest contribution to the soil quality index, and total N had the lowest weighting.

**Table 3:** Type of scoring curves, the parameters of non-linear and linear equations, and calculated weights for the minimum data set.

Indicator	Scoring curve	Non-linear		Linear		Weight
		Mean (*x*_*0*_)	Slope (b)	*y*_*max*_	*y*_*min*_	
Silt	Less is better	31.956	2.5	54.48	17.41	0.159
N	More is better	0.210	− 2.5	0.32	0.10	0.146
P	More is better	1.077	− 2.5	1.59	0.79	0.164
LAP	More is better	8.088	− 2.5	14.13	4.67	0.181
AP	More is better	93.071	− 2.5	106.72	72.75	0.158
Vector length	Less is better	1.505	2.5	1.68	1.31	0.189

### Accuracy verification of the MDS

**Figure 3:**
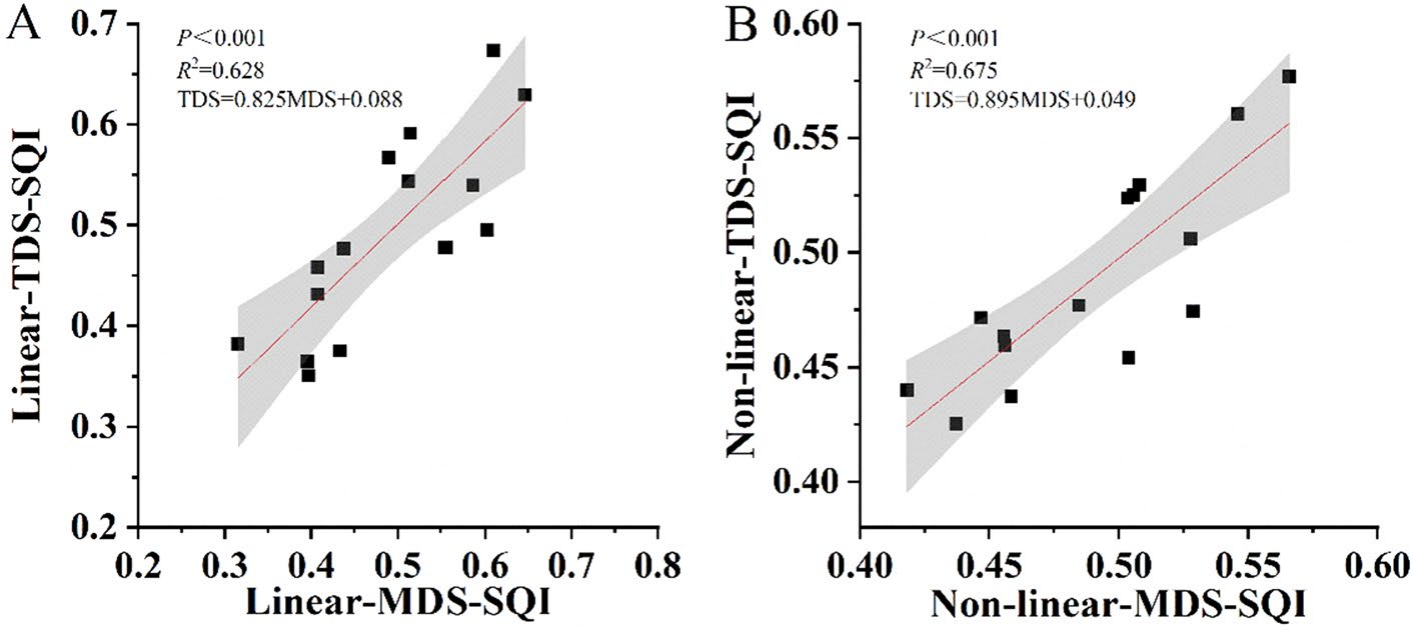
Linear regression analysis between MDS and TDS for linear and non-linear models. Linear-TDS-SQI: Linear soil quality index of the total data set, Linear-MDS-SQI: Linear soil quality index of the minimum data set, Non-linear-TDS-SQI: Non-linear soil quality index of the total data set, Non-linear-MDS-SQI: Non-linear soil quality index of the minimum data set. (**A**): The fitting curve of linear MDS and TDS, (**B**): the fitting curve of non-linear MDS and TDS.

The rationality verification of the MDS evaluation index system was an important part of SQI evaluation in the Yimeng Mountain Area, China (Fig. 3). The TDS and MDS of linear and non-linear exhibited a significant positive correlation (*P* < 0.001), the correlation coefficients determined using the non-linear method (*R*^2^ = 0.675) were higher and more accurate than those determined using the linear method (*R*^2^ = 0.628), indicating that the results of the two methods were in good agreement. Overall, the MDS method, like the TDS method, can be used as a feasible way of assessing soil quality.

### Soil quality index and analysis of contribution rate

**Figure 4:**
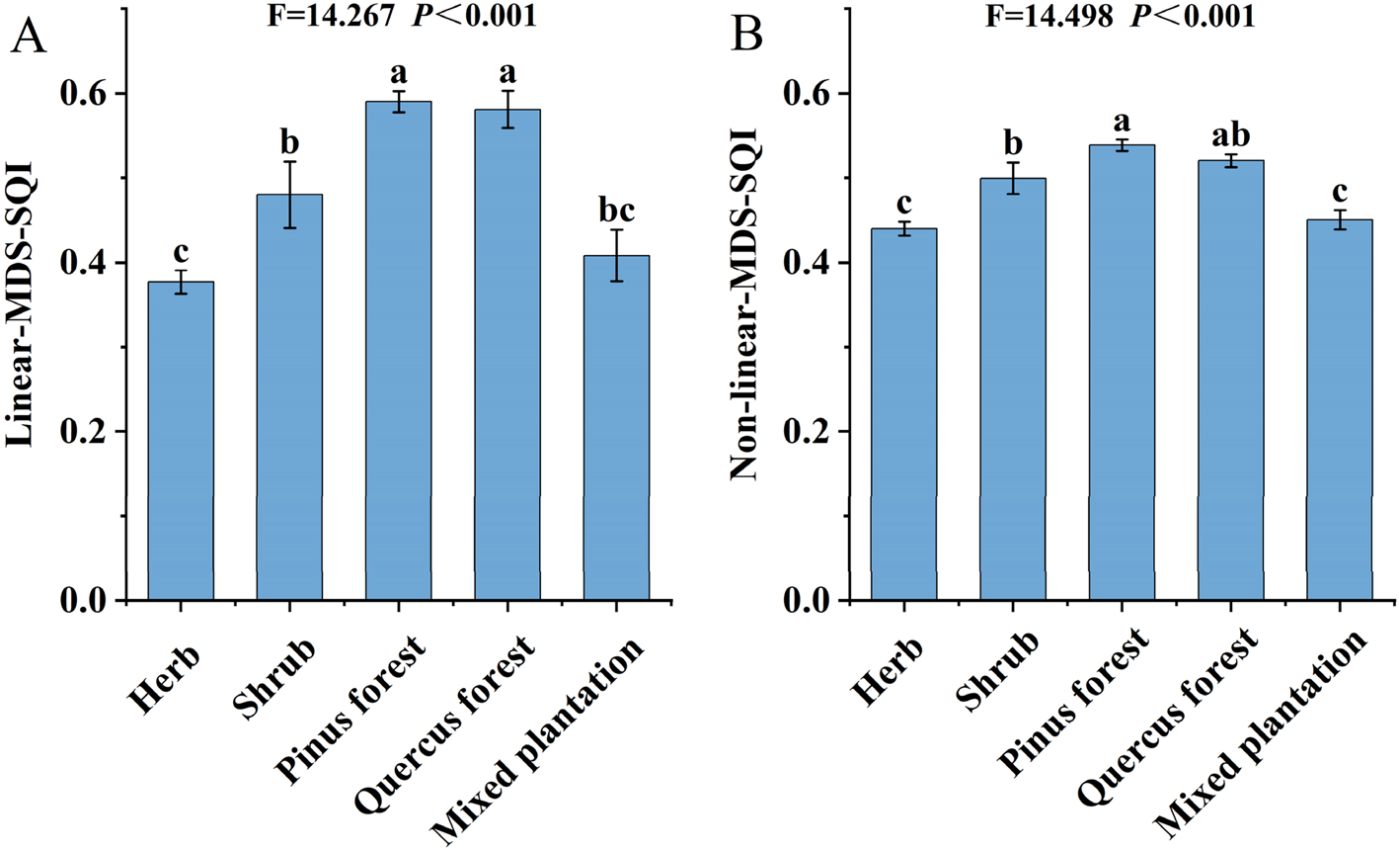
Characteristics of SQI under Yimeng Mountain area, SQI: Soil quality index. Linear-TDS-SQI: Linear soil quality index of the minimum data set (**A**), Non-linear-MDS-SQI: Non-linear soil quality index of the minimum data set (**B**).

The soil quality index (SQI) of the *Pinus* forest was higher than that of the other communities, and the mixed forest was similar to the herb in the linear and non-linear models (Fig. 4). The SQI values derived with the linear MDS method ranged from 0.37 to 0.59, the SQI values calculated by the non-linear MDS method ranged from 0.44 to 0.54. The SQI values based on the MDS method of linear and non-linear under different communities showed the same ordering from highest to lowest. The comparative SQI using linear- MDS-SQI Eq. (6) and non-linear- MDS-SQI Eq. (7) methods can be described as follows:

6 $$\begin{aligned} {\text{Linear - MDS - SQI}} = & \left( {0.{159} \times S_{{L({\text{Silt}})}} } \right) + \left( {0.{146} \times S_{{L({\text{N}})}} } \right) + \left( {0.{159} \times S_{{L({\text{P}})}} } \right) \\ & \quad + \left( {0.{165} \times {\text{S}}S_{{L({\text{LAP}})}} } \right) + \left( {0.{182} \times S_{{L({\text{AP}})}} } \right) + \left( {0.{189} \times {\text{S}}_{{L({\text{Vector length}})}} } \right) \\ \end{aligned}$$

7 $$\begin{aligned} & {\text{Non - linear - MDS - SQI}} \\ &\quad = 0.{159} \times \left( {{1}/\left( {{1} + S_{{NL({\text{Silt}})}} /{31}.{956}} \right)^{{{2}.{5}}} } \right) + 0.{146} \times \left( {{1}/\left( {{1} + S_{{NL({\text{N}})}} /0.{21}0} \right)^{{ - {2}.{5}}} } \right) \\ & \qquad + 0.{159} \times \left( {{1}/\left( {{1} + S_{{NL({\text{P}})}} /{1}.0{77}} \right)^{{ - {2}.{5}}} } \right) + 0.{165} \times \left( {{1}/\left( {{1} + S_{{NL({\text{LAP}})}} /{8}.0{89}} \right)^{{ - {2}.{5}}} } \right)\\ & \qquad + 0.{182} \times \left( {{1}/\left( {{1} + S_{{NL({\text{AP}})}} /{93}.0{71}} \right)^{{{2}.{5}}} } \right) + 0.{189} \times \left( {{1}/\left( {{1} + S_{{NL({\text{Vector length}})}} /{1}.{5}0{5}} \right)^{{{2}.{5}}} } \right) \\ \end{aligned}$$

The specific contribution of each linear MDS indicator to the SQI (Table 3) showed that AP had the highest contribution to the SOI of herbs. In contrast, the contribution was lower in the mixed plantation than in the other communities. Soil total N and P contributions were lower in the *Quercus* forest than in other communities. The specific contribution of each non-linear MDS indicator towards the SQI (Table 4) showed that six indicators contributed like to the SQI, and LAP had the lowest contribution towards the SQI of herbs. The soil total N and P contributions were lower in the *Quercus* forest, similar to the linear trend. N had the lowest contribution to the SOI of the mixed plantation.

**Table 4:** Relative contributions of selected soil indicators to SQI under different communities.

Model	Study site	Percentage of indicators (%)					
		Silt	N	P	LAP	AP	Vector length
Linear-MDS-SQI	Herb	10.11	28.69	15.18	0.74	43.66	1.62
	Shrub	17.41	24.52	3.12	13.37	32.12	9.46
	*Pinus* forest	17.87	18.84	22.60	7.08	12.18	21.43
	*Quercus* forest	21.38	3.32	2.86	27.16	25.96	19.31
	Mixed plantation	32.46	3.45	16.43	7.64	0.64	39.39
Non-linear-MDS-SQI	Herb	10.88	21.06	17.91	8.09	23.38	18.68
	Shrub	14.16	19.41	11.52	17.16	20.17	17.58
	*Pinus* forest	16.16	17.23	19.98	12.74	15.26	18.63
	*Quercus* forest	19.53	6.15	11.23	25.00	19.25	18.84
	Mixed plantation	24.10	6.28	18.49	13.27	14.31	23.55

*Linear-MDS-SQI* linear soil indicators contribution of the minimum data set, *Non-linear-MDS-SQI* non-linear soil indicators contribution of the minimum data set.

### Limiting soil indicators for soil quality

**Figure 5:**
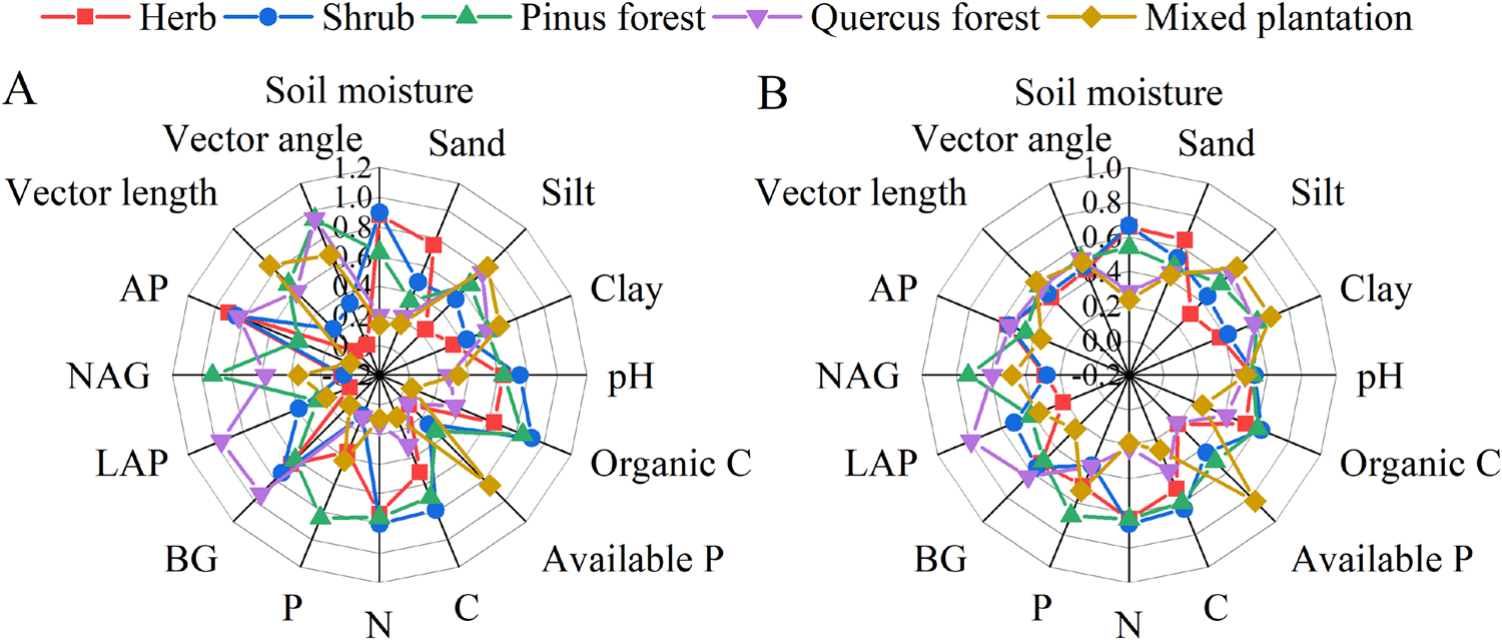
The limiting factors of soil quality index for SQI. (**A**): limiting factors of linear soil quality index for SQI, (**B**): limiting factors of non-linear soil quality index for SQI.

Scores of all soil parameters were plotted in a radar diagram to explore the limiting soil indicators for the linear-SQI and non-linear-SQI (Fig. 5). When lines under different communities crossed the axes, scores of each indicator were projected on the web. Crossing points located on the edge of the web indicated better soil quality, and crossing points near the centre of the web represented worse soil quality. According to the comprehensive analysis of the radar chart of the scores of the two evaluation indicators, silt, available P, LAP, NAG, vector length and vector angle were the major factors influencing herbs; available P, P, NAG, vector length and vector angle were the major factors influencing shrubs; available P, LAP, AP were the main influencing factors of *Pinus* forest; soil moisture, available P, C and N were the major factors influencing *Quercus* forest; and soil moisture, pH, organic C, C, N, BG, LAP and AP were the factors influencing the mixed plantation. The results indicated that combined with the proportion of each element in the minimum data set, soil quality was relatively good in the *Pinus* forest, and the performance results of the influencing factors were similar in linear and non-linear models. The mixed plantation was affected by soil texture, moisture and nutrients, but organic C and C influence were important reasons for the lower soil quality compared to other communities.

## Discussion

### Applicability of MDS indicators

The MDS for screening of the soil quality index indicators can reduce time and economic costs, and it is also the most widely used model for soil quality evaluation^33,34^. PCA combined with the norm value for MDS screening, effectively prevents the lack of important indicators in principal component screening^35^. In this study, 16 primary indicators were selected, 6 indicators of silt, total P, total N, LAP, AP and vector length were screened by MDS, and 62.5% of the indicators were screened and filtered, simplifying the evaluation of the soil quality index. The screening results included three aspects of physics, chemistry, and biology (Table S1), the indicators were more representative. The significant positive correlation of the linear model MDS and TDS (*R*^2^ = 0.628, *P* < 0.001), and significant positive correlation of the non-linear MDS and TDS (*R*^2^ = 0.675, *P* < 0.001), the correlation index can be used as the minimum data set determination index and the MDS method with similar accuracy to TDS method^13,24^. Thus, MDS reduces the number of indicators, and can be an effective replacement for the TDS method to evaluated soil quality of arid and barren mountainous.

The predecessors used the MDS to screen the index mostly including soil moisture, pH, silt, total N, total P, organic C, and available P^13,14,36^. In our study, silt, total N, and total P were retained in the MDS and were consistent with the results of previous studies^36^. However, pH, soil moisture, organic C, and available P were more researched and not selected, the selection criterion of the smallest data set was within 10% of the largest data in the group, and there was no correlation^37^. The pH, soil moisture and organic C were the same as the selected total N in the first group, and the norm values of soil moisture (2.001), pH (1.664) and organic C (2.014) compared with the maximum N (2.154) were 7.10%, 22.74% and 6.50%, respectively (Table 1). Soil moisture and organic C were correlated with N, which needed to be eliminated. The available P was the same as the selected LAP in the second group. The available P norm value (1.775) was within 10% of LAP (1.838) in this group, while was correlated with LAP, AP (1.803) was within 10% of LAP and not correlated, AP and LAP were selected as the MDS indicators. The vector length of enzymes in this article could be used to indicate the nutrient metabolism of microorganisms^38^, and represent the biological properties of the soil. The norm value of vector length was highest in the fourth group, therefore, we confirmed that vector length can be one of the MDS indicators in forest regions.

### Soil quality assessment and influencing factors

Research has shown that the applicability of linear and non-linear index evaluation is related to location and index complexity. Yuan et al.^23^ evaluated soil quality in rice-crayfish farming in the Jianghan Plain using a linear method, which was simple to use and almost does not require prior knowledge of the system; only the threshold of each indicator needs to be understood, and the score of each indicator was determined by observation^39,40^. Qiu et al.^41^ evaluated soil quality in *Larix principis-rupprechtii* plantations in North China using a non-linear method, this method requires a full understanding of the characteristics of each indicator, and the operation is complicated^42^. While Andrews et al.^34^ believed that the non-linear index evaluation was more suitable for soil quality evaluation, the difference between treatments was smaller than linear. In this study, the MDS of these two assessment methods were significantly correlated with the TDS (*P* < 0.001, Fig. 3), and the SQI obtained of five communities by non-linear scoring model was in line with that obtained by linear, but the details were slightly different (Fig. 4), the F-values of SQI obtained using the non-linear scoring method (F = 14.498) in the ANOVA were all greater than the linear scoring method (F = 14.267). The result indicated that non-linear evaluation can balance the proportion of each index evaluation compared with linear evaluation^13^, thus, the non-linear method could better distinguish the soil quality of the five communities of arid and barren mountainous ^43^, the non-linear scoring method is more representative of the system function than the linear scoring function^42^.

The soil quality index was calculated using the integrated quality index Eq. ^44^. The results showed that the soil quality was relatively higher in *Pinus* forest than in other communities in the assessment (Fig. 4), the SQI was lower and similar to that of the herb and mixed plantation. This result was inconsistent in that the soil quality gradually increased from grass to shrub to tree in karst^24^. In this study, the forest was divided into artificial forest plantations for approximately 30 years. Our previous study indicated that the soil moisture was the lowest in mixed plantations^45^, which indicates a moderately dry period of soil. Appropriate soil gravel content increases soil ventilation and water permeability, while more sand content and less clay content will accelerate soil erosion^46^. The sand was highest and soil moisture was the lowest in mixed plantations, which affects soil water availability and increase ecological vulnerability^45,47^. Another consideration is that according to the Niche Theory^48^, the diameter at breast height and *Quercus* of the mixed plantation was lower than that of the *Quercus* pure forest, and the growth of the *Pinus* was not as good as that of the* Pinus* pure forest. The height of *Quercus* trees was higher than that of *Pinus*, which results in insufficient light and affects the photosynthesis of *Pinus* (Table S1). In the mixed plantation, there was intraspecific and interspecific competition between *Quercus* and *Pinus*, and understorey shrubs^49^, which was affected by various factors such as soil moisture soil structure, organic C, vegetation type and microbial nutrient metabolism^12,50,51^, the poor growth and lower SQI of the mixed plantation.

Radar plots of soil parameter scores can indicate limiting indicators of soil quality^24^. In this study, the influencing factors on the soil quality of herbs, shrubs, *Pinus* forests and *Quercus* forests were soil available P, LAP, NAG, and AP related to N- and P- acquiring enzymes. While the influencing factors in the soil quality of the mixed plantation were organic C, total C and BG, these were different from those of the other communities. The accumulation rate of organic C is different in the soil environments of different communities^52,53^. The soil organic C (13.60 mg g^−1^), total C (21.13 mg g^−1^) and BG (26.21 nmol g^−1^ h^−1^) of the mixed plantation were lower than those of other communities^45^, which are key indicators of soil quality^54^. The organic C and C of the mixed plantation were lower, resulting in a reduction in the carbon source provided and significantly affecting soil carbon sequestration and nutrient cycling^55^. This was also an important reason for the lower soil quality of the mixed plantation. Therefore, the soil quality was affected by the soil chemical properties and enzymes of different communities.

## Conclusions

The improvement of soil quality in arid and barren mountainous areas plays an important role in enhancing the carbon sequestration capacity of forest communities. To evaluate the soil quality of five communities in arid and barren mountainous in the Yimeng Mountain area, China. Sixteen soil physical, chemical, and biological properties were determined, and significant correlations were shown between them. The MDS of six indicators (silt, total N, total P, LAP, AP. vector length) was established in this research for soil assessment. The SQI results showed that *Pinus.* forest was higher than those of the other communities, and the soil quality index of mixed plantation was lower and similar to the herb community. Soil chemical properties and enzymes influence the soil quality of different communities. Unlike other communities, the soil quality in the mixed plantation was affected by total C and organic C. Hence, in the subsequent production and management of forest stands, we should focus on maintaining the stability of the ecosystem, and strengthening the transformation of the mixed plantation.

### Supplementary Information

Supplementary Tables.

## Data Availability

The data and materials presented in this study are available on reasonable request from Chuanrong Li.
