# Supplementary material for: Assessment of soil quality in an arid and barren mountainous of Shandong province, China
Source: Sci Rep. 2023 Nov 15;13:19966. doi: 10.1038/s41598-023-46136-6 (PMC10652006; doi:10.1038/s41598-023-46136-6)
Supplement: Supplementary file 1 — Supplementary Tables. [file 41598_2023_46136_MOESM1_ESM.docx]

Table S1 Descriptive statistics of measured soil properties of five communities

| Type | Soil indicator | Unit | Range |
| --- | --- | --- | --- |
| Physical properties | Moisture | % | 4.015-11.375 |
|  | Sand | % | 42.41-82.22 |
|  | Silt | % | 17.41-54.48 |
|  | Clay | % | 0.07-3.10 |
| Chemical properties | pH | Unitless | 4.19-5.10 |
|  | Organic C | mg·g^-1^ | 13.11-26.77 |
|  | Available P | mg·kg^-1^ | 1.04-5.39 |
|  | Total C | % | 1.81-4.60 |
|  | Total P | mg·g^-1^ | 0.79-1.59 |
|  | Total N | % | 0.09-0.32 |
| Biological properties | BG | nmol·g^-1^·h^-1^ | 26.64-52.76 |
|  | LAP | nmol·g^-1^·h^-1^ | 4.67-14.13 |
|  | NAG | nmol·g^-1^·h^-1^ | 7.55-18.31 |
|  | AP | nmol·g^-1^·h^-1^ | 72.75-106.72 |
|  | Vector length | Unitless | 1.31-1.68 |
|  | Vector angle | ° | 53.86-61.23 |

Table S2 The forests growth features of the sampling sites, Q.: *Quercus acutissima* forest, P.: *Pinus thunbergii* forest.

| Study site | Height (m) | Diameter at breast height (cm) | Crown East West × North South(m×m) |
| --- | --- | --- | --- |
| *Quercus* forest | 9.68±0.28 | 19.24±0.22 | (3.97±0.09)×(3.39±0.05) |
| *Pinus* forest | 8.76±0.23 | 18.17±0.20 | (3.95±0.06)×(3.46±0.25) |
| Mixed plantation | *Q.*9.64±0.28  *P.*8.21±0.21 | *Q.*17.74±0.31  *P.*17.25±0.25 | *Q.*(3.46±0.22)×(2.94±0.07)  *P.*(2.71±0.08)×(2.33±0.16) |
